# Supplementary material for: Seminoma and Embryonal Carcinoma Footprints Identified by Analysis of Integrated Genome-Wide Epigenetic and Expression Profiles of Germ Cell Cancer Cell Lines
Source: PLoS One. 2014 Jun 2;9(6):e98330. doi: 10.1371/journal.pone.0098330 (PMC4041891; doi:10.1371/journal.pone.0098330)
Supplement: File S1 — ZIP file containing DMRforPairs output for significant regions. Please start from the html files. (ZIP) [file pone.0098330.s008.zip › figures/5322.pdf]

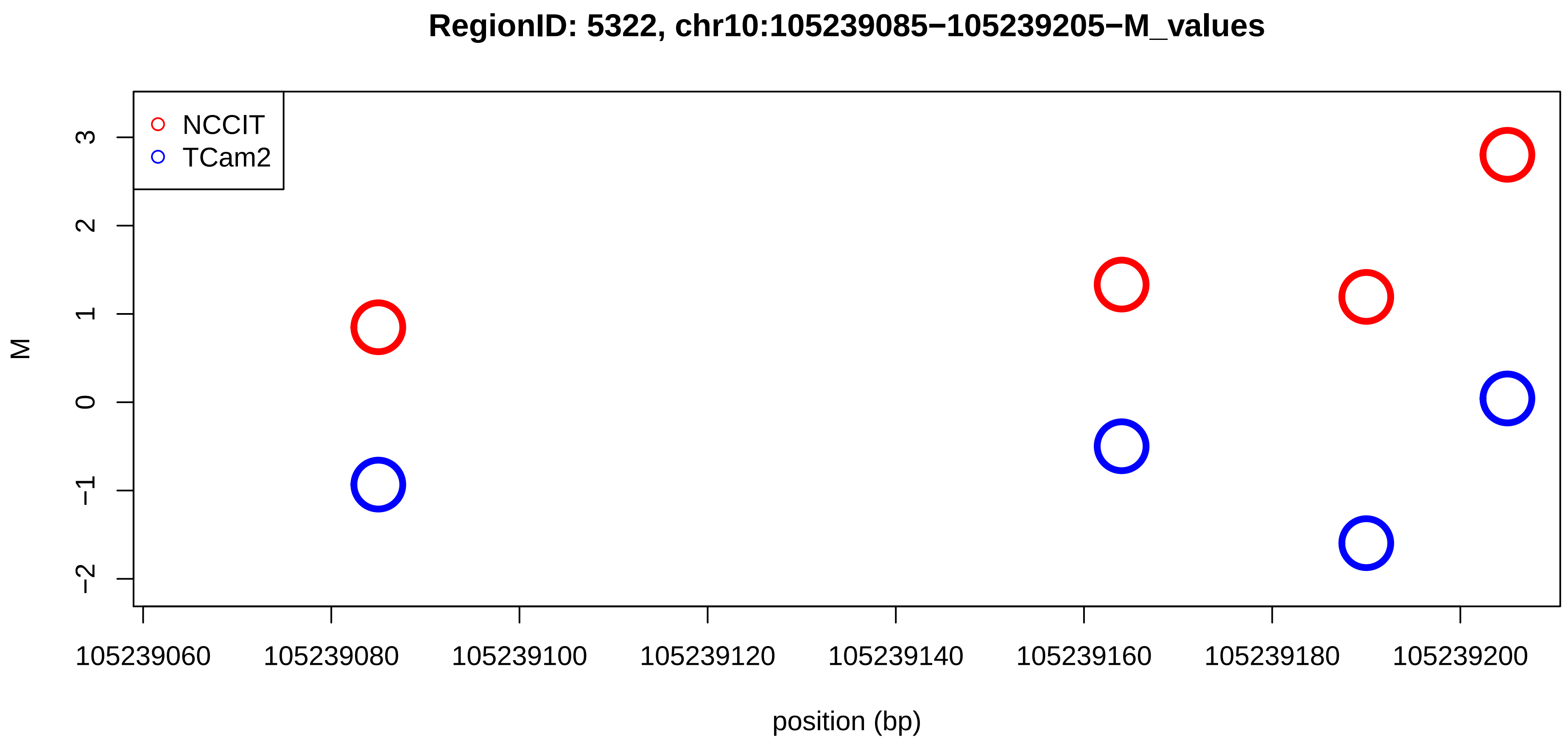
RegionID: 5322, chr10:105239085−105239205−M_values
NCCIT
TCam2
M
position (bp)
3
2
1
0
−1
−2
105239060
105239080
105239100
105239120
105239140
105239160
105239180
105239200

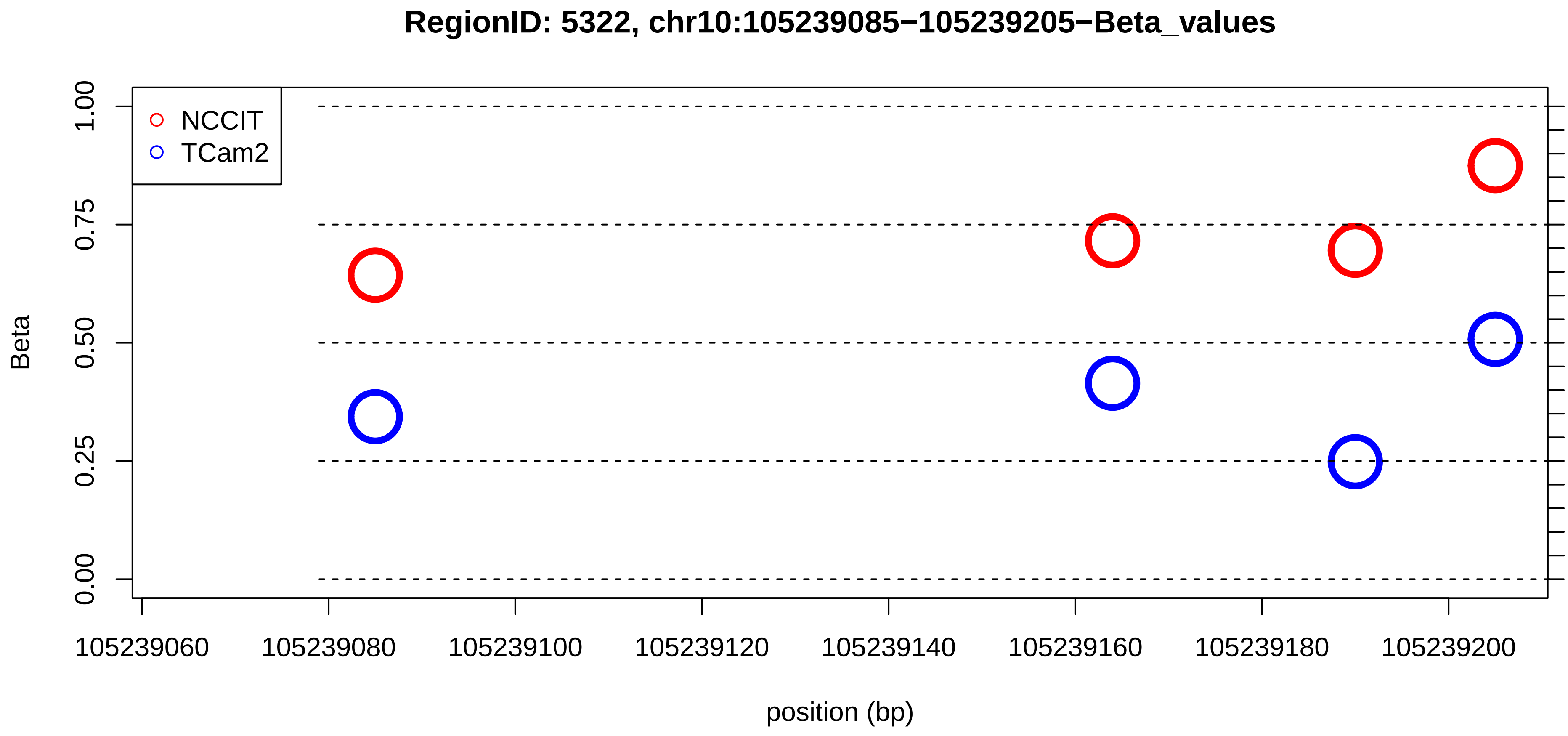
RegionID: 5322, chr10:105239085−105239205−Beta_values
NCCIT
TCam2
Beta
position (bp)
1.00
0.75
0.50
0.25
0.00
105239060
105239080
105239100
105239120
105239140
105239160
105239180
105239200
